# A Mechanism of Reducing Methane Production During Sewage Sludge Composting by Adding Urea

**DOI:** 10.3390/toxics12120895

**Published:** 2024-12-10

**Authors:** Ke Zhang, Haopeng Guo, Yujing Liang, Fuyong Liu, Guodi Zheng, Jun Zhang, Aihua Gao, Nan Liu, Chuang Ma

**Affiliations:** 1 School of Material and Chemical Engineering, Zhengzhou University of Light Industry, Zhengzhou 450000, China; 2017093@zzuli.edu.cn (K.Z.); haopengguo@126.com (H.G.); yujingliang3322@163.com (Y.L.); liu.fyong@gmail.com (F.L.); pirola2008@zzuli.edu.cn (N.L.); 2 Collaborative Innovation Center of Environmental Pollution Control and Ecological Restoration, Zhengzhou 450000, China; 3 Institute of Geographic Sciences and Natural Resources Research, Chinese Academy of Sciences, Beijing 100101, China; 4 College of Environmental Science and Engineering, Guilin University of Technology, Guilin 541004, China; 5 Zhongyuan Ecological Environment Technology Innovation Center (Henan) Co., Ltd., Zhengzhou 450000, China

**Keywords:** sewage sludge composting, urea, methane emission, bacterial communities

## Abstract

The study of the effect of the mechanism of urea addition to sewage sludge and sawdust-composting substrates on methane production is still limited. In the present study, the systematic investigation of the effect of urea addition (0.18, 0.9 and 1.8 kg) on methane production is discussed through the dynamics of physical properties, enzymes, and the microbial community during composting. The results showed that high urea addition (1.8 kg) suppressed methane production, with a lower rate and a shorter duration of warming in the thermophilic phase, but significantly enhanced cellulase activity, urease, and peroxidase, and promoted the degradation of organic carbon, as well as the loss of nitrogen. A high addition of urea stimulated the growth and reproduction of *Sinibacillus*, *Pseudogracilibacillus*, *Sporosarcina*, and *Oceanobacillus*. The random forest model indicated that the top six independent determinants of CH_4_ emissions were *Methanobacterium*, temperature, organic matter (OM), *Methanospirillum*, and NH_4_^+^-N. Furthermore, structural equation modeling displayed that NH_4_^+^-N, O_2_, and pH were the main physicochemical properties affecting CH_4_ emissions. *Methanobacterium*, *Methanosarcina*, and *Methanosphaera* were the main archaea, and *Bacillaceae* were the main bacteria affecting CH_4_ emissions. This study provides new insights and a theoretical basis for optimizing urea addition strategies during composting.

## 1. Introduction

Globally, more than 1.37 million tons of sewage sludge (SS) are produced annually [1]. This sludge is laden with pollutants, such as heavy metals, pathogenic microorganisms, and exogenous compounds, posing significant risks to public health by contaminating soil, air, and water resources. Composting is an effective method of decomposing OM (e.g., animal manure, food waste, etc.) from sewage sludge into stable end products [2]. However, this process is not without its drawbacks. The composting process generates greenhouse gases (GHGs), notably CH_4_ and CO_2_, through aerobic and anaerobic reactions linked to carbon metabolism [3], resulting in secondary pollution and diminishing the environmental advantages of composting [4]. Since the 1850s, the global average surface temperature has risen by 1.09 °C, primarily due to the escalating concentration of GHGs, particularly CO_2_, CH_4_, and N_2_O (Intergovernmental Panel on Climate Change (IPCC), 2021) [5]. Although CH_4_ accounts for approximately 20% of global warming, it possesses a higher warming potential and exerts a more potent short-time warming effect than CO₂ [6]. During composting, CH_4_ emission is second only to CO_2_, with the total carbon (TC) content of the emitted CH_4_ potentially reaching 2–3% of the TC content in the composted material [7]. Consequently, it is crucial to mitigate the production of CH_4_ in the composting process to minimize its environmental impact.

Urea is a prevalent additive to modulate the carbon to nitrogen ratio in composting technology [8] by affecting the composting system’s temperature, pH, and other physicochemical properties. The production of CH_4_ during composting is closely related to the activities of microorganisms, and the addition of urea as a nitrogen source can potentially alter these activities, including those of methanogenic bacteria, subsequently affecting CH_4_ production. While urea may stimulate CH_4_ emissions under certain conditions, some studies have indicated that a high concentration of nitrogen fertilizer could suppress the formation and release of CH_4_ in the soil. This inhibition may be attributed to the fact that, in a nitrogen-rich environment, certain methanogenic competitors, such as *Methanosphaera*, *Bacillaceae* and *Methanogens*, consume hydrogen and OM in the soil, thereby inhibiting the growth and activity of methanogens and reducing CH_4_ emissions [9]. However, the mechanism of urea on CH_4_ emissions during compositing is not yet clear.

It has been shown that a variety of physicochemical factors, including temperature, pH, water content, oxygen concentration, and the OM decomposition rate, play a pivotal role in CH_4_ production and emission during the composting process [10]. Urea, as a nitrogen-rich additive, effectively modulates the carbon to nitrogen balance within the compost, thereby facilitating the breakdown of organic compounds and elevating the compost temperature [11]. Furthermore, the decomposition of urea enriches ammonia-nitrogen into the compost, which in turn alters the pH levels, rendering the environment more alkaline [12]. These shifts can significantly influence the metabolic functions of microorganisms, especially by disrupting the equilibrium between methanogenic and CH_4_ oxidizing bacteria [13]. Consequently, a comprehensive examination of the impact of urea on the physicochemical properties of compost is crucial for understanding its role in the dynamics of CH_4_ emission.

Incorporating urea into compost not only refines its physical and chemical attributes but also significantly influences on the enzymatic activities within the compost matrix [14]. The ammonia released from urea decomposition can activate hydrolytic enzymes, which are essential for the breakdown of OM. These enzymes include cellulases and proteases, both playing pivotal roles in this process [15]. This enhancement in enzyme activity accelerates the degradation of organic materials. Moreover, the introduction of urea may modulate the activities of enzymes involved in redox reactions, such as dehydrogenase [16]. This modulation can regulate the compost’s redox environment, subsequently impacting the processes of methanogenesis and CH_4_ oxidation. Therefore, a detailed examination of how urea modulates enzyme activities is essential for unraveling the underlying mechanisms that govern its influence on CH_4_ emissions.

CH_4_ is generated through the anaerobic metabolic activities of archaeal community during the composting process [17]. CH_4_-producing archaea, known as methanogens, play a pivotal role in achieving efficient and balanced waste decomposition [7]. Both bacteria and the archaeal community are integral functional communities within composting [18]. However, the majority of previous research has concentrated on the dynamics of bacterial communities, often overlooking the archaeal communities. Tracking the shifts in both bacterial and archaeal community structures can yield a wealth of information on microbial succession throughout the composting process. The composition of the microbiota during composting is influenced by the combination of raw materials used, yet our understanding of how these materials impact the diversity and ecosystem functioning of various bacterial populations remains limited.

Environmental factors, including the C/N ratio, OM content, and temperature, are essential for successful composting [19]. However, the impact of different urea ratios on CH_4_ production and archaeal community dynamics during sewage sludge (SS) composting has not been systematically studied. In the present study, we conducted composting of SS and sawdust (m/m) with varying amounts of urea, ensuring that microorganisms had access to a consistent carbon source but varying nitrogen sources. The aims of this study were (1) to investigate the effect of different levels of urea addition on CH_4_ emissions during SS composting, (2) to examine how these additions affect the bacterial and archaeal communities during composting, and (3) to elucidate the mechanism by which urea reduces CH_4_ production during sewage sludge composting. This study provides novel insights into strategies for mitigating CH_4_ emissions.

## 2. Materials and Methods

### 2.1. Compost Materials and Environmental Design

Dewatered sewage sludge was collected from the Wulongkou municipal wastewater treatment plant (WWTP) (Zhengzhou, China). The sawdust was composed of pine wood particles (1–2 mm). The sewage sludge and sawdust were homogenized to obtain a ratio of 3:1 (*w*/*w*, fresh weight). Piles A, B, and C were supplemented with urea at dosages of 0.18, 0.9, and 1.8 kg, respectively. The total weight of each pile was approximately 130 kg. The initial physicochemical properties of the raw materials and each pile are shown in Table 1.

### 2.2. Composting System and Sampling

Composting was conducted in three separate but identical piles for 30 days. Each pile had a height of 120 cm, an inner diameter of 60 cm, and an effective volume of 280 L. The piles were made from polyethylene and covered with a 3 cm thick rubber board for thermal insulation. A removable lid with a small hole (2 cm in diameter) was placed on the top and uniformly distributed holes (1 cm in diameter) were installed at the bottom of each pile. The open space at the bottom of each pile was 40%, and leachates were not generated. A ventilator was installed at the bottom of each pile and aeration was provided at 0.5 L·min^−1^·kg^−1^ (0–2 d) and 1.87 L·min^−1^·kg^−1^ (on a dry weight basis, 3–10 d). During the composting process, aeration and aeration intervals can control microbial activity, promote decomposition of OM, avoid accumulation of harmful substances, improve composting efficiency and quality, and reduce energy consumption [20]. The ventilator was run for 3 min and then stopped for the next 17 min. Temperature sensors were incorporated into a metal bar, which was positioned at vertical intervals of 20 cm, 40 cm, and 60 cm along its length. Moreover, a suction pump was fitted in each pile at a height of 50 cm and connected to a shunt device to divide the air into two portions: one portion was directed to an oxygen sensor [21] and the other portion was directed to a storage gas bag. Using a pump, about 1 L of the air collected in the storage gas bag, after having repeatedly been reversed and mixed thoroughly, was transferred to an aluminum foil gas bag for the determination of CH_4_ concentrations [22]. O_2_ and CH_4_ were measured for 20 min, which was the period of one ventilation cycle.

Compost was sampled on 1, 3, 10, 20, and 30 days after installation. Three samples of approximately 500 g were collected from each pile at a depth of 50 cm on each sampling day and divided into two subsamples. One set of subsamples was used to determine physicochemical properties and enzymatic activities, the other set was homogenized and refrigerated at −80 °C for subsequent DNA analysis.

### 2.3. Measuring of Samples

The moisture content of the raw materials and composting mixtures was ascertained by subjecting the samples to a drying regimen at 105 °C for a period of 24 h within an oven [23]. The OM content was determined by assessing the mass loss of each airdried sample upon combustion in a muffle furnace at 550 °C for 24 h [24]. A 1:5 (*w*/*v*) mixture of the wet sample and water was shaken for 30 min to allow for equilibration, after which the pH was determined using an E-201-C combination pH meter (Lei-ci, Shanghai, China). The NH_4_^+^-N content was colorimetrically determined using the KCl extraction indigo phenol blue method. The CH_4_ concentration within the air enclosed in the aluminum foil bag was measured using gas chromatography (TianMei 7900, Shanghai, China) [25]. The total organic carbon (TOC) was oxidized by 0.8 (1/6) mol L^−1^ potassium dichromate (K_2_Cr_2_O_7_) with external heating [26]. The total nitrogen(TN) in the compost samples were detected by a Vario EL elemental analyzer (Elemental Vario MICRO, Hanau, Germany). Brassica chinensis was used for the germination index (GI), and the procedures and calculation method have been provided in a previous study [26].

### 2.4. Enzymatic Activity Assays

The activities of five extracellular enzymes (cellulase, urease, protease, arylsulphatase, and peroxidase) were determined at each sampling time. Cellulase, urease and protease enzyme activities were determined using the methods described by Tabatabai et al. [27]. Cellulase activity was determined colorimetrically using 3,5-dinitrosalicylic acid, while the activity of urease was determined using colorimetry of indophenol blue. Protease activity was activity was measured colorimetrically using ninhydrin. The activities of arylsulphatase (ARS) and peroxidase (POD) were monitored by a Thermo Scientific Multiskan (FC, Thermo Fisher Scientific, Waltham, MA, USA) according to methods detailed by Galgani [28].

### 2.5. DNA Analysis

To identify the bacterial and archaeal diversity during the composting process, the microbial DNA of each pile on days 1, 3, 10, 20, and 30 were extracted using a FastDNA^®^ SPIN Kit for soil (MP Bio, Santa Ana, CA, USA) according to the manufacturer’s protocol. DNA concentration and purity were determined with a nanodrop 2000 ultraviolet-visible spectrophotometer (Thermo Fisher Scientific, Waltham, MA, USA). The V3-V4 hypervariable regions of the bacterial 16S rRNA gene were amplified with the primers 338F (ACTCCTACGGGAGGCAGCAG) and 806R (GGACTACHVGGGTWTCTAAT). The V4–V5 hypervariable regions of the archaeal gene were amplified with the primers 524F10extF (5′-TGYCAGCCGCCGCGGTAA-3′) and Arch958RmodR (5′-YCCGGCGTTGAVTCCAATT-3′) [29], using the thermocycler PCR system (GeneAmp 9700, ABI, Los Angeles, CA, USA). The PCR reactions were conducted as follows: denaturation for 3 min at 95 °C, 27 cycles of 30 s at 95 °C, annealing for 30 s at 55 °C, elongation for 45 s at 72 °C, and extension for 10 min at 72 °C. PCR reactions were performed in triplicate using a 20 μL mixture of 4 μL 5 × FastPfu Buffer, 2 μL of 2.5 mM dNTPs, 0.8 μL of each primer (5 μM), 0.4 μL of FastPfu Polymerase, and 10 ng template DNA. The PCR amplicons were isolated from a 2% agarose gel and subsequently subjected to purification utilizing the AxyPrep DNA Gel Extraction Kit from Axygen Biosciences, Union City, CA, USA, and their concentrations were determined employing QuantiFluor™-ST (Promega, Madison, WI, USA), strictly adhering to the manufacturer’s prescribed protocol.

### 2.6. Statistical Analysis

The data are presented as mean values with standard deviations. Figures were generated using OriginPro (Version 9.4). The least significant differences among the mean values during composting were calculated at a probability level of *p* < 0.05 using the SPSS software (Version 24.0). Spearman correlation analysis via R (pheatmap package) software was used to analyze enzymatic activity and bacterial genera. The random forest model (RF) and structural variance model (SEM) were analyzed by the R package lavaan to verify the direct and indirect effects of the studied variables on the rate of CH_4_ production. We used a non-significant chi-square test (*p* > 0.05) a high goodness-of-fit index (*GFI* > 0.90), and low root mean square errors of approximation (*RMSEA* < 0.05) to show a good fit for SEMs.

## 3. Results and Discussion

### 3.1. Changes in Physicochemical Properties During Composting

#### 3.1.1. Temperature

**Figure 1:**
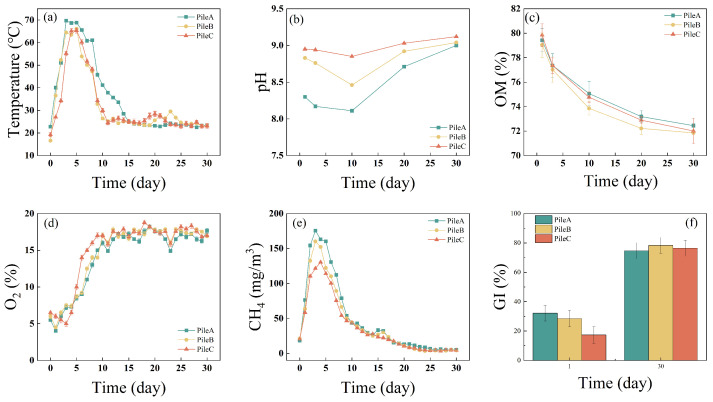
Changes in (**a**) Temperature, (**b**) pH, (**c**) OM, (**d**) O_2_, (**e**) CH_4_ and (**f**) GI during composting.

Temperature is an important indicator during the composting process, reflecting the degradation of organic material and the efficiency of composting [23,30]. The three piles underwent the three classic temperature-defined composting phases, mesophilic, thermophilic, and cooling phases, as depicted in Figure 1a. The peak temperature occurred on day 3 for pile A and on day 5 for piles B and C. Piles A and B maintained a high-temperature period of at least 55 °C for six days, whereas pile C only sustained this temperature for five days. It can be inferred that, compared to pile A, the addition of 0.9 kg (pile B) and 1.8 kg (pile C) of urea seemed to suppress the compost’s heating rate, with pile C also exhibiting a shorter duration of the thermophilic phase relative to piles A and B. These observations align with the findings of Sun et al. [31], which suggests that urea addition can suppress the average temperature increase rates and the maximum temperature achieved. Furthermore, the extended periods of high temperature in piles A and B are likely sufficient to ensure the complete destruction of pathogens, thereby meeting the requirement for harmlessness [32].

#### 3.1.2. Change in pH, OM, O_2_ and CH_4_

The pH is a critical factor influencing microbial activity during the composting processes [33]. The pH values for all piles exhibited a consistent trend (Figure 1b), with a slight decrease from 8.3–8.95 to 8.11–8.85 over the first ten days. This initial decline is mainly attributed to the production of alkaline ammonia, which subsequently caused the pH to rise to 9–9.12, maintaining alkaline levels by day 30 [34]. Notably, the pH of pile C was significantly higher than that of pile B, and pile B’s pH was considerably higher than pile A (*p* < 0.05). The low pH of pile A is possibly due to the lower concentration of urea compared to the other piles. The degradation of urea likely produced NH_4_^+^ ions [35], resulting in a more alkaline environment.

OM is an important energy source for microbes [36]. The OM content of the three piles changed similarly (Figure 1c) and decreased throughout the composting process, which is in line with the findings of Zhao et al. [34]. The initial OM content of the three piles (80%) was significantly higher than the final OM content (72%). By the end of the composting period, OM decreased by 8.80%, 9.10%, and 9.82% for each treatment, respectively. This uniform degradation of OM suggests that the three piles, containing equal amounts of SS and sawdust, were not significantly affected by the addition on OM content. The OM degradation rate was highest during the first three days, possibly because microorganisms initially targeted easily degradable OM, followed by more recalcitrant components like cellulose and lignin [37]. These observations indicate that the addition of urea promoted the degradation of OM in the compost, with the most significant degradation occurring at the 1.8 kg urea addition level (pile C).

Oxygen concentration(OC) is a limiting factor on microbial metabolism during SS composting [38] because parts of the compost matrix are anaerobic, specifically in the large compost particles [4]. The change of OC across the three treatments was similar (Figure 1d). On day 1, pile C exhibited a significantly higher OC than piles B and A (*p* < 0.05), with piles A and B reaching their lowest OC levels of 4.0% and 4.5%, respectively. This is likely due to the substantial O_2_ consumption by microorganisms during the initial stages of OM degradation [21]. On day 3, pile C’s OC dropped to its lowest point of 5.0%, remaining significantly higher than that of piles B and A (*p* < 0.05) from day 5 onwards. After the fourth day, the O_2_ concentration of the three piles began to rise, reaching peak levels of 16.0%, 17.0%, and 17.0% day 10, respectively. The decrease in microbial activity due to lower temperatures and reduced OM led to a natural slowdown in decomposition, marking the transition to the compost maturation phase [39], indicating a significant improvement in anaerobic conditions and the predominance of aerobic conditions.

CH_4_ is primarily produced under anaerobic conditions [40]. The rapid decomposition of easily degradable OM in the early stages of composting leads to a swift decline in pile O_2_ concentration (Figure 1e) [41], causing a sharp initial increase in CH_4_ emissions from all treatments, followed by a gradual decrease (Figure 1e). The CH_4_ emissions from each pile were mainly produced during the mesophilic phase and the early stage of the thermophilic phase [42]. Between day 1 and day 3, the CH_4_ emissions of all treatments were in the order of pile A > pile B > pile C. This could be attributed to pile A having a higher temperature (Figure 1a) and microbial activity, leading to an insufficiency of O_2_ (Figure 1e). The low CH_4_ emissions in pile C could be due to the inhibition effect of high ammonia levels, generated by the hydrolysis of urea, which can inhibit the growth of methanogenic bacteria [43]. The CH_4_ emissions from each pile increased with rising composting temperature and decreasing in O_2_ content in the compost, peaking between days 3 and 4. As composting continued, the size of anaerobic zones within the pile gradually decreased, resulting in reduced CH_4_ production [21]. Our results indicate that the addition of 1.8 kg of urea suppressed CH_4_ emissions during the composting process compared to 0.18 kg and 0.9 kg, which is consistent with previous research [6].

#### 3.1.3. Changes in TN, NH_4_^+^-N, TOC Contents and C/N

**Figure 2:**
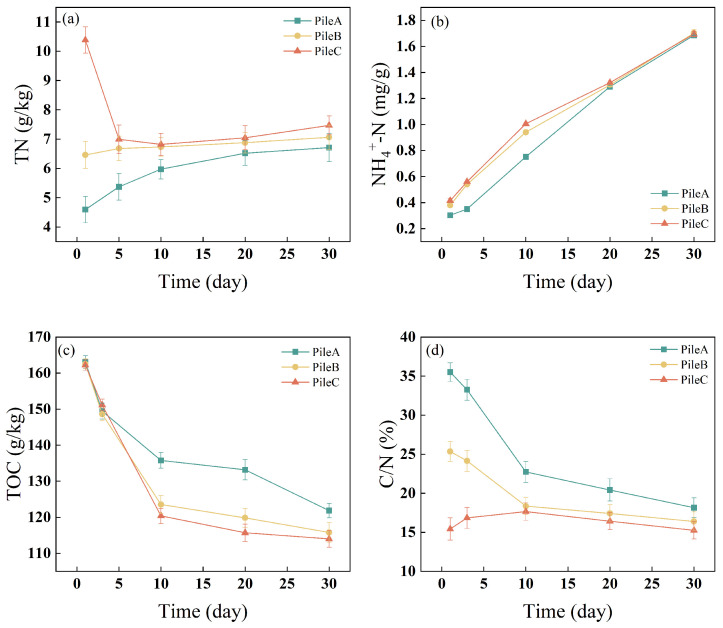
Changes in (**a**) Temperature, (**b**) pH, (**c**) OM and (**d**) O_2_ during composting.

In the sludge composting process, nitrogen loss in the form of ammonia constitutes a significant proportion of the TN of the raw material [30]. The TN content in piles A and B first decreased and then increased, but the TN content in pile C showed a gradual decline, consistently remaining higher than that in piles A and B (Figure 2a). During the mesophilic phase, the decomposition of urea led to the production and release of substantial amounts of NH_3_, resulting in a decrease in the TN content [30]. Throughout the composting process, the difference in TN content among the three piles gradually diminished, persisting until the end of composting. Denitrification may be the main cause of nitrogen loss during the composting process [31]. After the twentieth day, there was no significant difference in TN content among the three piles.

NH_4_^+^-N is the main source of inorganic nitrogen for microbial utilization during composting [44]. The NH_4_^+^-N contents of all three piles continually increased throughout the composting process (Figure 2b), starting at the lowest (0.3, 0.37, and 0.41 mg/g, respectively) on day 1 and peaking (1.68, 1.70, and 1.69 mg/g, respectively) on day 30. During the first 20 days, piles B and C had significantly higher NH_4_^+^-N contents than pile A (*p* < 0.05), but at later stages of composting, the NH_4_^+^-N contents of the three piles did not differ significantly. This may be due to the substantial addition of urea in pile B and pile C [44]. The degradation of urea, uric acid, and proteins during composting likely caused the increased NH_4_^+^-N contents. These results differ from those of previous studies, in which the NH_4_^+^-N content decreased during composting [18], possibly due to the urea addition in this experiment, which led to an increase in NH_4_^+^-N content. These results are similar to those of Ren et al. [45], who observed an increase in NH_4_^+^-N content during composting, reaching a maximum at the end of the process. This could be attributed to increased ammonification and inhibited nitrification at higher temperatures [44].

TOC and TN are the main chemical parameters used to evaluate the potential of a successful composting process [46]. Mature compost usually has a low and stable C/N ratio, typically between 10 and 20. The TOC in the three piles generally declined during the whole composting process, with no significant difference among the three piles on day 3 (Figure 2c). From the tenth day onwards, the difference in TOC content among the three piles gradually increased. From the tenth day to the end of composting, the TOC content in pile A was significantly higher than that in piles B and C. This indicates that the degradation of OM mainly occurs in the thermophilic phase (Figure 1c) and that a relatively high amount of urea added (0.9 kg and 1.8 kg) is more conducive to the microbial degradation of organic carbon [46]. This relationship may be due to the relative insufficiency of nitrogen sources when urea is added at low levels (0.18 kg), which limits microbial activity.

The C/N ratio, a result of changes in TC and TN during composting, is one of the most important parameters in composting. During the composting process, the C/N ratio in piles A and B showed a decreasing trend (Figure 2d), this is agreement with the results of Zhang et al. [44]. In contrast, the C/N ratio of pile C showed a trend of increasing first and then decreasing. The C/N ratio in pile A was always higher than the C/N ratio in pile B, and the C/N ratio in pile B was always higher than the C/N ratio in pile C. At the end of composting, C/N ratio in pile C was lower than that in pile A and pile B, indicating that highly spiked urea supply may be beneficial for changes in organic components [44].

#### 3.1.4. Changes in GI

The seed GI is a crucial parameters for assessing the toxicity of compost products to plants and for evaluating the quality of the compost [26]. A higher GI value indicates lower toxicity to plants and superior product quality [26]. Prior to composting, the GI values for piles A, B, and C were 32.15%, 28.46%, and 17.32%, respectively (Figure 1f). After composting, these values increased to 74.63%, 78.35%, and 76.42% for piles A, B, and C, respectively. The GI indexes after composting were significantly higher than the GI indexes before composting. The increase in GI during composting process is likely due to the reduction in concentrations of NH_4_^+^-N, volatile fatty acids (VFAs), and other phytotoxic substance in the compost samples, thereby reducing their toxic effect [31]. Consequently, the addition of urea had no significant effect on the maturity of the final compost product.

### 3.2. Changes in Enzymatic Activities

#### 3.2.1. Cellulase Activity

**Figure 3:**
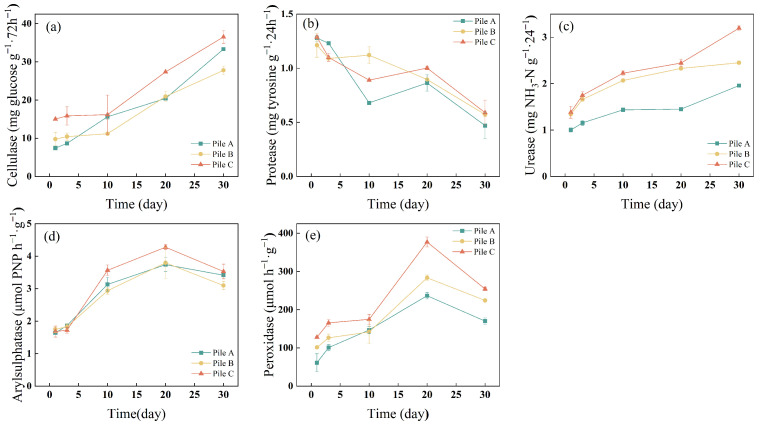
Evolution of enzyme activities of different piles during the composting process. Enzyme legends: (**a**) cellulase, (**b**) protease, (**c**) urease, (**d**) arylsulfatase, (**e**) peroxidase. Note: significance is considered at *p* < 0.05.

Cellulase activity, which gradually increased with composting time, had a similar trend among the piles (Figure 3a). The highest cellulase activity, at 33.33, 27.78, and 36.52 (mg glucose g^−1^·72 h^−1^) for piles A, B, and C, respectively, was observed on day 30. Similar trends were also observed by Tiquia [23]. Cellulase activities are pivotal bioindicators of cellulose degradation, typically augmenting during the maturation phase of composting as the more readily degradable organic matter has been predominantly metabolized by microbes in the initial stages of the composting process [10]. In pile C, the cellulase activities were significantly higher than those in piles A and B, except on day 10 (*p* < 0.05). The possible reason for this is that a higher urea addition is beneficial to the growth of cellulolytic microorganisms, which may stimulate the release of cellulase for the degradation of cellulose to glucose, which serves as a carbon source for other microorganisms [47]. Therefore, higher urea addition (1.8 kg) conditions stimulate cellulase production, and are more conducive to cellulose degradation.

#### 3.2.2. Protease Activity

Protease activity exhibited a general downward trend during the whole composting process, and there was no significant difference among the three piles (Figure 3b). The highest protease activity, at 1.28, 1.21, and 1.29 (mg tyrosine g^−1^·24 h^−1^), in piles A, B, and C, respectively, was observed on day 3. On day 30, the protease activity decreased to a minimum of 0.47, 0.57, and 0.589 (mg tyrosine g^−1^·24 h^−1^) in piles A, B, and C, respectively. The gradual decline in protease activity during composting might be related to the bioavailability of proteins in the initial composting materials. Specifically, easily degradable OM, such as carbohydrates and proteins, is metabolized by microorganisms in the early stage of composting. Thus, microorganisms secrete more protease at this time. Then, protease activity wanes as proteins are increasingly consumed, culminating in the lowest levels at the conclusion of the composting period. However, the increased addition of urea did not exert a significant influence on the dynamics of protease activity during the sewage sludge composting process.

#### 3.2.3. Urease Activity

Urease is involved in the hydrolysis of urea to ammonium and carbon dioxide [46]. The urease activity in piles gradually increased with composting time, reaching its highest levels, 1.9581, 2.4507, and 3.1956 (mg NH_4_^+^-N g^−1^·24 h^−1^) in piles A, B, and C, respectively, on day 30 (Figure 3c). The urease activity in pile C was significantly higher than that in piles A and B during the whole composting process (*p* < 0.05). This suggests that a high addition of urea (1.8 kg) fostered an increase in microbial metabolism that was resilient to elevated temperatures, accelerated the mineralization and decomposition rate of nitrogen-containing OM, and consequently heightened urease activity [10]. It has been suggested that higher urea addition leads to higher ammonia production [43], which was confirmed by the production of urease in the present study. The results indicated that higher urea addition (1.8 kg) could not only enhance composting efficiency, but also increase urease activity, which might promote the production of ammonia gas during the thermophilic phase of composting [45]. As a result, balancing compositing efficiency and odor production is challenging, and needs further study in terms of regulating the urea addition to achieve high compost quality [43].

#### 3.2.4. Arylsulphatase Activity

Arylsulfatase (ARS) catalyzes the detachment of sulfate groups from organic compounds, and its enzymatic activity is correlated with the formation of humus [48]. ARS activity had similar trends among the piles (Figure 3d); it gradually increased from day 3 to 20 and then began to decrease. The urea addition had no significant effect on the activity of ARS. The activity of ARS in pile C was relatively higher than those in piles A and B from days 10 to 20, and the activity of ARS in pile A was relatively lower than those in piles B and C on day 10. The results indicated that a higher urea addition (1.8 kg) stimulated ARS activity, which in turn increased H_2_S production during the anaerobic mesophilic phase of the composting process [49]. Therefore, it is necessary to supply oxygen in the initial stage of composting [49], especially under higher urea addition (1.8 kg) conditions.

#### 3.2.5. Peroxidase Activity

Peroxidase (POD) is the most extensively researched extracellular enzyme associated with white-rot fungi and can oxidize lignin polymers [30]. The change trend of POD activity was similar among the three treatments during the whole composting process (Figure 3e), with two peaks on days 5 and 20, but the POD activity under a high urea addition was generally higher than that under a low urea addition, especially on days 20 to 30, when the differences among the piles were significant (*p* < 0.05). The variation in POD activity indicated that a higher urea addition was more conducive to POD secretion, and promoted the degradation of OM in compost because POD for lignin degradation is usually produced by fungi and is active during the cooling and maturing period of compost [23].

### 3.3. Evolution of Bacterial and Archaeal Communities

#### Effects of Different Initial Urea Additions on the Bacterial and Archaeal Community Structure

**Figure 4:**
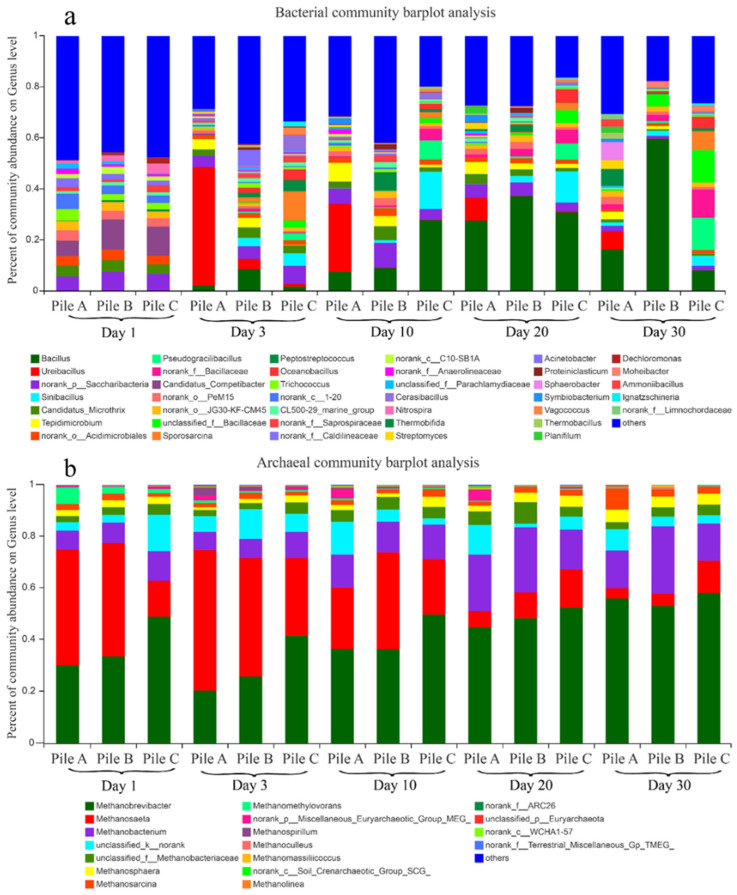
Changes in bacterial (**a**) and archaeal (**b**) community composition in different piles at the genus level during the composting process.

The coverage index (>0.99) of the three piles indicated that most of the microorganisms had been detected in the samples [50]. Variations in initial urea additions resulted in distinct physicochemical conditions and bacterial growth environments [19], which in turn affected the diversity of bacteria and archaea within the different treatments. As shown in Figure 4a, *Candidatus_Competibacter* and *norank_1–2others* dominated the compost mixture on day 1. During the thermophilic phase, *Bacillus* showed a continuous increasing trend, from 2.2%, 8.6%, and 1.8% on day 3 to 27.7%, 37.2%, and 30.9% on day 20 in piles A, B, and C, respectively. On day 30, the proportion of *Bacillus* in piles A and C decreased to 16.3% and 8.2%, respectively, while it continued to rise to the maximum level of 59.7% in pile B. Bacillus was the dominant bacteria in the thermophilic phase and in the cooling phase for all three piles, especially pile B. *Ureibacillus* reached a maximum level of 46.4% on day 3 in pile A and then gradually decreased to 7.1% on day 30. This result indicated that *Ureibacillus* dominated the bacterial community during the thermophilic phase in pile A, suggesting that the conditions in pile A were conducive to the proliferation of *Ureibacillus*. In pile C, the proportion of *Sinibacillus* increased from 4.9% on day 3 to 14.6% on day 10, and then decreased to 3.9% on day 30. *Sinibacillus* was one of the dominant genera in the thermophilic phase of pile C, suggesting that high urea addition (pile C) was beneficial to the growth and reproduction of *Sinibacillus*. Similarly, high urea addition (pile C) was also beneficial to the growth of *Pseudogracilibacillus*, *Sporosarcina*, and *Oceanobacillus*. As thermotolerant members of the *Bacillaceae* family, *Sinibacillus* species have demonstrated a robust resistance to radiation, chemicals, heat, and drought, endowing them with the versatility required to thrive in a range of harsh environmental conditions [10].

As shown in Figure 4b, throughout the composting process, the archaeal communities in the three piles were dominated by (in decreasing order) *Methanobrevibacter* (20.3–58% of archaea), *Methanosaeta* (4–54.5%), *Methanobacterium* (7–26%), and *unclassified_k_norank* (1.3–14%). The *Methanobacterium* and *Methanobrevibacter* populations increased during composting, while the *Methanosaeta* population decreased. The trends for *Methanobrevibacter* and *Methanosaeta* populations were similar in all three piles. *Methanobrevibacter* was the dominant archaea on the first composting day (contributing 30, 33, and 48% of the archaea in piles A, B, and C, respectively). The contributions of *Methanobrevibacter* decreased up to day 3 of the composting process (to 20, 25.7, and 41.5% in piles A, B, and C, respectively), and then increased and reached a maximum on day 30 (55.8, 52.9, and 58% in piles A, B, and C, respectively). *Methanobrevibacter*, which is intricately associated with CH_4_ production, was consequently the predominant archaea throughout the composting process, while the higher urea addition had increased relative abundance of *Methanobrevibacter*.

The contributions of *Methanosaeta* to the archaeal community first increased and then decreased in all three piles, and were 44.7, 43.8, and 13.8% in piles A, B, and C on the first day, respectively; 54.4, 45.7, and 30% on day 3, and reached a minimum of 4, 4.7, and 12.5% on day 30. *Methanosaeta* was the dominant archaea during the thermophilic phase and likely the main source of CH_4_ because emissions mainly occurred during this phase (Yuan et al., 2016). This was also the case during the mesophilic phase. However, a high urea addition decreased the proportion of *Methanosaeta*. The *Methanobacterium* population in all three piles followed a similar trend to that of the *Methanobrevibacter* population. The contributions of *Methanobacterium* to the archaea in piles A, B, and C were 7.3, 8, and 11.4%, respectively, on day one; 7, 7.4, and 10.5% on day 3; and 14.6, 26.2, and 14.3% on day 30. This contribution increased markedly during the late thermophilic phase and the cooling phase, indicating that *Methanobacterium* was the dominant archaea in the late composting period and was not strongly affected by the initial urea additions. The contribution of *unclassified_k_norank* was markedly higher for pile A than for piles B and C during the intermediate and later phases of the thermophilic phase and the cooling phase, indicating that a low urea addition increased the proportion of *unclassified_k_norank*.

The impact of varying initial urea additions on CH_4_ production during sludge composting was investigated. Cellulase and arylsulfatase were the main enzymes affecting the composting process, and both were negatively correlated with CH_4_. The inhibition of CH_4_ emission by high urea addition was mainly achieved by changing the relative abundance of dominant bacteria and dominant archaea, which altered the activities of the main functional enzymes. NH_4_^+^-N, O_2_, pH was the main physicochemical property affecting CH_4_ emission, Methanobacterium, Methanosarcina, and Methanosphaera were the main archaea affecting CH_4_ emissions, and Bacillaceae were the main archaebacteria affecting CH_4_ emissions. We conclude that urea supplementation at a dose of 1.8 kg (pile C) is effective in reducing CH_4_ emissions.

### 3.4. Effects of Physicochemical Properties, Enzymes, and Microbial on CH_4_

**Figure 5:**
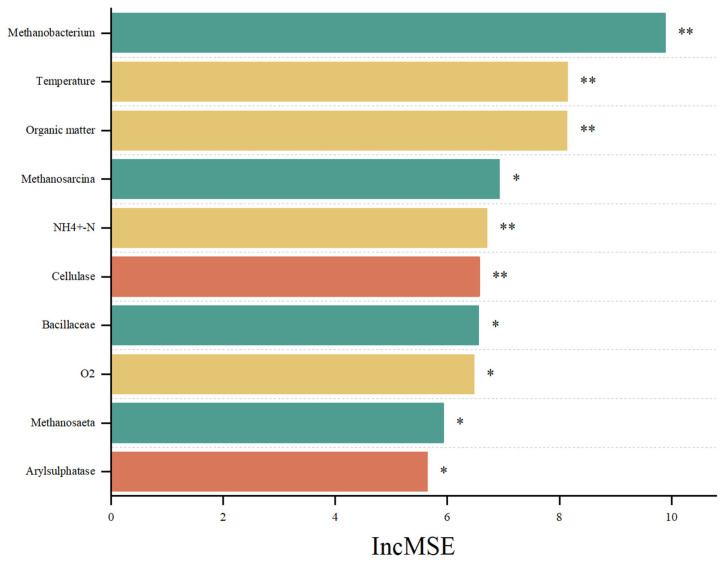
Random forest models (RF) quantifying the contribution of urea coupled with exogenous factors to microbial community structure, networks, and environmental genes. * *p* < 0.05, ** *p* < 0.01.

From Figure 5, the random forest (RF) model quantified the combined effects of physicochemical properties, enzymes, and microorganisms on CH_4_ emissions. The top six independent determinants of CH_4_ emissions were *Methanobacterium*, temperature, OM, *Methanospirillum*, and NH_4_^+^-N. Among the explanatory variables, the main physicochemical property determinants of CH_4_ emissions were temperature, OM, NH_4_^+^-N, and O_2_, and the main enzymes determining CH_4_ emissions were cellulase and arylsulphatase. The main archaea determining CH_4_ emissions were *Methanobacterium*, *Methanosarcina*, and *Methanosphaera*, and the main bacteria determining CH_4_ emissions were *Bacillaceae*.

**Figure 6:**
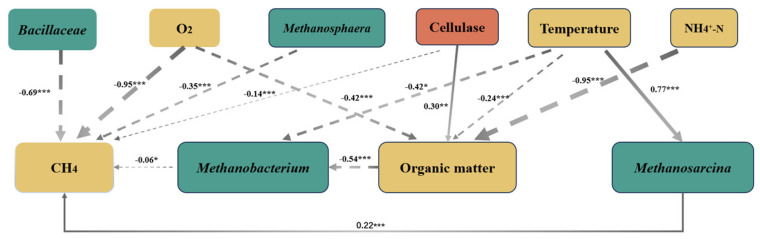
The structural equation model showed the direct and indirect effects of association of CH_4_ with cellulases, physicochemical properties, *Methanobacterium*, and *Methanosarcina*. The continuous arrow and the dotted arrow indicate positive and negative relationships, respectively. Asterisks denote significant effect (*, *p* < 0.05; **, *p* < 0.01; ***, *p* < 0.001).

It can be seen from Figure 6 that cellulase is the main enzyme that affects the composting process [51]. Figure 6 shows that cellulase is positively correlated with O_2_, OM, and NH_4_^+^-N, but negatively correlated with CH_4_, temperature, and *Bacillaceae*. Cellulase breaks down carbohydrate polymers to release nutrients from organic compounds and is an enzyme that plays an important role in the carbon conversion cycle, with hydrolyzed products including glucose [7]. Given that the concentration of NH_4_^+^-N exhibits a strong positive correlation with the presence of cellulose (*p >* 0.05), the relationship between cellulase and dominant bacteria was changed by the high level of EPI (pile C: 1.8 kg).

From Figure 6, it can be seen that temperature, O_2_, and NH_4_^+^-N were significantly negatively correlated with *Methanosphaera* (*p* < 0.05), while *Methanosphaera* was significantly negatively correlated with CH_4_ (*p* < 0.05) and temperature was significantly positively correlated with *Methanosarcina* (*p* < 0.05). These correlations indicated that temperature, O_2_ and NH_4_^+^-N had a negative effect on *Methanosphaera*, temperature had a negative effect on *Methanosarcina*, and *Methanosphaera* had a negative effect on CH_4_ production. The archaeal community structure was similar in pile A and pile B during the neutrophilic (day 1), thermophilic (day 3), and maturation (day 30) phases of composting, and significantly different from pile C. NH_4_^+^-N, O_2_ and CH_4_ were the main physicochemical properties affecting the community structure of compost bacteria and archaea. Yang et al. [16] found that adding urea to compost materials resulted in lower CH_4_ emissions and significant N_2_O losses. Szanto et al. [4] suggested that composting reduces CH_4_ production by altering the physicochemical properties of compost.

## 4. Conclusions

The effect of different initial urea additions on CH_4_ production during sludge composting was investigated. Cellulase and arylsulfatase were the main enzymes affecting the composting process, and both were negatively correlated with CH_4_. The inhibition of CH_4_ emission by a high urea addition was mainly achieved by changing the relative abundance of dominant bacteria and dominant archaea, which altered the activities of the main functional enzymes. NH_4_^+^-N, O_2_ and temperature were the main physicochemical properties affecting CH_4_ emission; *Methanobacterium*, *Methanosarcina*, and *Methanosphaera* were the main archaea affecting CH_4_ emissions; and *Bacillaceae* were the main archaebacteria affecting CH_4_ emissions. We conclude that urea supplementation at a dose of 1.8 kg (pile C) is effective in reducing CH_4_ emissions. The findings of this study evaluated the potential effectiveness of urea in suppressing CH_4_ emissions throughout the composting process. This research presents a pioneering strategy for mitigating CH_4_ emissions during composting, and sheds new light on the influence of urea on microbial activities and the composting process as a whole.

## Figures and Tables

**Table 1:** Physicochemical properties of raw materials used in this study.

Parameters	Sewage Sludge	Sawdust	Pile A	Pile B	Pile C
pH	8.12	5.55	8.30	8.83	8.95
Moisture content	80.52%	8.23%	51.34%	51.11%	51.09%
Organic matter	43.39%	97.66%	84.42%	84.04%	84.84%
Total organic carbon	28.11%	57.82%	36.06%	36.57%	35.96%
Total nitrogen	2.05%	0.41%	1.03%	1.41%	2.34%
C/N ratio	13.32	140.30	35.51	25.34	15.42

## Data Availability

The raw data supporting the conclusions of this article will be made available by the authors upon request.
